# High Level Expression and Purification of Recombinant Proteins from *Escherichia coli* with AK-TAG

**DOI:** 10.1371/journal.pone.0156106

**Published:** 2016-05-23

**Authors:** Dan Luo, Caixia Wen, Rongchuan Zhao, Xinyu Liu, Xinxin Liu, Jingjing Cui, Joshua G. Liang, Peng Liang

**Affiliations:** 1 Department of Biochemistry & Molecular Biology, School of Life Sciences, Sichuan University, Chengdu, China; 2 Clover Biopharmaceuticals, Chengdu, China; 3 GenHunter Corporation, Grassmere Park, Nashville, United States of America; University of Queensland, AUSTRALIA

## Abstract

Adenylate kinase (AK) from *Escherichia coli* was used as both solubility and affinity tag for recombinant protein production. When fused to the N-terminus of a target protein, an AK fusion protein could be expressed in soluble form and purified to near homogeneity in a single step from Blue-Sepherose via affinity elution with micromolar concentration of P1, P5- di (adenosine—5’) pentaphosphate (Ap5A), a transition-state substrate analog of AK. Unlike any other affinity tags, the level of a recombinant protein expression in soluble form and its yield of recovery during each purification step could be readily assessed by AK enzyme activity in near real time. Coupled to a His-Tag installed at the N-terminus and a thrombin cleavage site at the C terminus of AK, the streamlined method, here we dubbed AK-TAG, could also allow convenient expression and retrieval of a cleaved recombinant protein in high yield and purity via dual affinity purification steps. Thus AK-TAG is a new addition to the arsenal of existing affinity tags for recombinant protein expression and purification, and is particularly useful where soluble expression and high degree of purification are at stake.

## Introduction

In the post-genomic era, functional studies of genes rely in part on the expression and characterization of protein products of interest. To this end, the concurrent use of fusion tags with DNA cloning technology has become a routine practice in recombinant protein expression and purification [1–4]. Although numerous affinity tags have been developed over the years to facilitate the expression and purification of recombinant proteins from *Escherichia coli* [5–10], Glutathione S-transferase (GST-Tag) [11], Maltose-binding protein (MBP-Tag) [12] and 6xHIS-Tag [13] remain the most popular methods of choice due to the commercially available expression vectors and their downstream purification systems.

All tags, whether large or small, can often impede upon the structure and functions of a target protein expressed and may need to be removed during or after purification [6, 10, 14, 15]. Thus, a specific proteolytic cleavage site is often introduced between the tag and the protein of interested to be expressed on an expression vector. Among many site specific proteases used for the cleavage of the tags’ off target proteins [7, 16, 17], thrombin is the most widely used due to its high target sequence-specificity and rarity found in natural proteins [18–21].

Despite the wide-spread use of these tailor-made expression vectors and purification strategies, frustrations often occur when a target protein is expressed either at low level, or as insoluble inclusion bodies [5, 22]. Although His-Tag may allow purification of insoluble proteins after complete unfolding with a denaturant, the yield of refolding and full recovery of biological functions of a recombinant protein may be less predictable [23]. In addition, depending on the level of expression, the yield and purity of a recombinant protein from a single affinity column can be far from perfect due to endogenous host cell proteins bound to the columns.

Adenylate Kinase (AK) catalyzes the enzymatic conversion of AMP to ADP using ATP: Mg^2+^ATP + AMP ↔ Mg^2+^ADP + ADP, and is an essential enzyme in all living organisms [24, 25]. Previous work from our laboratory as well as by others showed that AK from *Escherichia coli* could be expressed not only at high level in soluble and active form in bacteria, but also easily purified to near homogeneity in a single step involving the affinity elution of the enzyme bound to Blue-Sepharose beads with its transitional state substrate analog, Ap5A [25–30].

We envisioned that AK, when used as a protein fusion tag, may aid not only the soluble expression of a recombinant protein which could be easily verified by a sensitive enzymatic AK reaction, but also allow the recombinant protein to be purified in one step to a level unattainable by any previously published affinity tags. Here we demonstrate the proof of concept in an effort to express TNFα as an AK fusion protein. Moreover, by introducing a His-Tag at the N-terminus and a thrombin cleavage site at the C terminus of AK, the method we dubbed AK-TAG can also allow the expression and recovery of a fully functional cleaved recombinant protein such as TNFα in high yield and purity via dual affinity purification steps.

## Materials and Methods

### Chemicals

T4 DNA ligase, Taq polymerase, and restriction enzymes were obtained from Takara. Isopropyl-beta-D-thiogalactopyranoside (IPTG) was purchased from Merck. 3-(4,5-dimethyl-2-thiazolyl)-2,5-diphenyl-2-H-tetrazolium bromide(MTT), Fetal Bovine Serum (FBS), P1, P5- di(adenosine—5’) pentaphosphate (Ap5A), glucose, ADP, NADP+, hexokinase and glucose-6-phosphate dehydrogenase were from Sigma. Thrombin (Restriction Grade) was obtained from Millipore.

### Strains and plasmids

pQE32 (Qiagen) was propagated in *Escherichia coli* XL1-Blue (Qiagen). pAK-T4 and pKILLIN vectors were described recently[30, 31].

### Plasmid DNA constructs

pAK-TAG was essentially as described in the expression of AK-T4 DNA ligase fusion protein [30]. Thrombin cleavage site was then inserted into pAK-TAG at the end of the AK coding region before the multiple cloning sites using a linker composed of L-thrombin (5’ -GCTGGTGCCGCGTGGCAGCGGTAC-3’) and R-thrombin (5’-CGCTGCCACGCGGCACCAGCGACG-3’) primers. The resulting vector was designated as pAK-TAG-T. For the expression of AK-TNFα fusion proteins, DNA fragment encoding the mature human TNFα was amplified by PCR using L-TNFα (5’-GGGGTACCGTCAGATCATCTTCTCG-3’) and R-TNFα (5’-GGGAAGCTTTCACAGGGCAATGATCC-3’) and cloned into pAK-TAG and pAK-TAG-T vectors at KpnⅠand HindIII sites. All plasmid constructs were verified by DNA sequence analysis.

### Enzyme Assay for Adenylate Kinase (AK)

AK enzyme assay was carried out as previously described [26, 27]. The reaction mixture (50μl) consists of 10 mM glucose, 2 mM ADP, 0.5 mM NADP+, 1.2 units of hexokinase and 0.6 units of glucose-6-phosphate dehydrogenase (Sigma). The AK activity was calculated by the rate of increase in absorbance at 340 nm. The formation of 1μmol of ATP in one minute is defined as one unit of AK enzyme activity. Protein concentration was measured by BCA assay kit (Pierce), according to the manufacturer’s instructions.

### Expression of recombinant pAK-TNFα and pAK-T-TNFα in *Escherichia coli*

Cell growth and induction of expression was carried out as previously described [30]. Briefly, bacteria cells were grown in LB medium at 37°C until reaching 0.6 at OD_600_, 1mM IPTG was then added to induce the protein expression for 10 h at 30°C. After induction, cells were harvested by centrifugation and protein extracts were prepared by sonication in extraction buffer as previously described [27].

### Quantification of AK fusion protein expression by AK activity

The level of a soluble AK fusion protein expression was estimated by the fold of increase in the specific activity of AK over that of the host cell XL1-blue containing pQE32.

### Purification of AK-TNFα and AK-T-TNFα by Blue-Sepharose column

After IPTG induction, the cells were collected by centrifugation at 4000 g for 20 min at 4°C. The cell pellet was resuspended in 20 ml of buffer A (20 mM Tris-HCl, pH 7.4, 1 mM MgCl_2_) and then lysed by sonication. Cell debris was removed by centrifugation at 16900 g for 20 min at 4°C. Protein purification was carried out using an AKTA-Prime purification system (GE Healthcare). About 80 mg of cleared cell lysate was used as a starting material and passed through a 1 ml HiTrap Blue HP (Blue-Sepharose) column (GE Healthcare) pre-equilibrated with 20 Column volumes (CVs) of buffer A. After sample loading, 10 CVs buffer A was added to wash off weakly bound proteins until OD_280_ detection declined to near baseline. The AK-TNFα and AK-T-TNFα fusion protein was eluted with a 20 CVs buffer B (20 mM Tris-HCl, pH 7.4, 50μM Ap5A, 1mM MgCl_2_). The flow rate was maintained at 1 ml/min throughout the purification [26, 27].

### Purification of cleaved human TNFα by Ni-NTA Column

AK-T-TNFα eluted from Blue-Sepharose column was passed through a Ni-NTA column (GE Healthcare) which was pre-equilibrated with 15 CVs of buffer C (20 mM Tris-HCl, pH 7.4, 500 mM NaCl, 5 mM imidazole). After washing off weakly bound contaminating proteins with 15 CVs of buffer C until OD_280_ declined to near the baseline, another 15 CVs of buffer D (20 mM Tris-HCl, pH 8.4, 150 mM NaCl, 2.5 mM CaCl_2_) was passed through the column. In-column thrombin digestion was initiated after passing 1.5 ml thrombin (9U in buffer D) and kept at the room temperature for 20 h. The column was washed with 10 CVs of buffer D followed by elution of the cleaved human TNFα with 10 CVs of buffer C. The His-tagged AK was finally eluted with a 15 CVs linear-gradient of 5–500 mM imidazole in buffer E (20 mM Tris-HCl, pH 7.4, 500 mM NaCl, 500 mM imidazole).

### SEC-HPLC analysis of protein purity and molecular-weight

The purity of purified AK-TNFα and TNFα was evaluated on an Agilent 1260 HPLC using Size-Exclusion Chromatography (SEC-HPLC) with an analytic TSK G3000SWXL column (Tosoh). Phosphate Buffered Saline (PBS) was used as the mobile phase with OD_280_ nm detection over a 20 min period at a flow rate of 1 ml/min. The protein molecular weight was estimated based on retention time using the protein size standard (BioRad).

### Cytotoxicity assay

The biological activity of TNFα was measured in vitro according to established method [32]. Briefly 1×10^4^ cells of L929 cells (ATCC) was seeded into each well of a 96-well cell plate with RPMI-1640 (Hyclone) containing 10% FBS. After 24 h, actinomycin D was added to the medium at 1 μg/ ml together with various concentrations of either TNFα (R&D), cleaved TNFα or AK-TNFα. After 18 hours of cell culture at 37°C, 0.5 mg/mL MTT was added to each well followed by a 4 h of incubation at 37°C. Then the media in the wells were carefully discarded and 100μl of dimethylsulfoxide (DMSO) was added into each well to dissolve Formazan crystal.The absorbance at 490 nm was recorded.

### Plasmid sequence accession numbers

The full sequence data of pAK-TAG and pAK-TAG-T expression vectors have been deposited in GenBank under the accession numbers, KX147098 and KX147099, respectively.

## Results

### High-level expression of recombinant AK fusion proteins in soluble form

**Fig 1:**
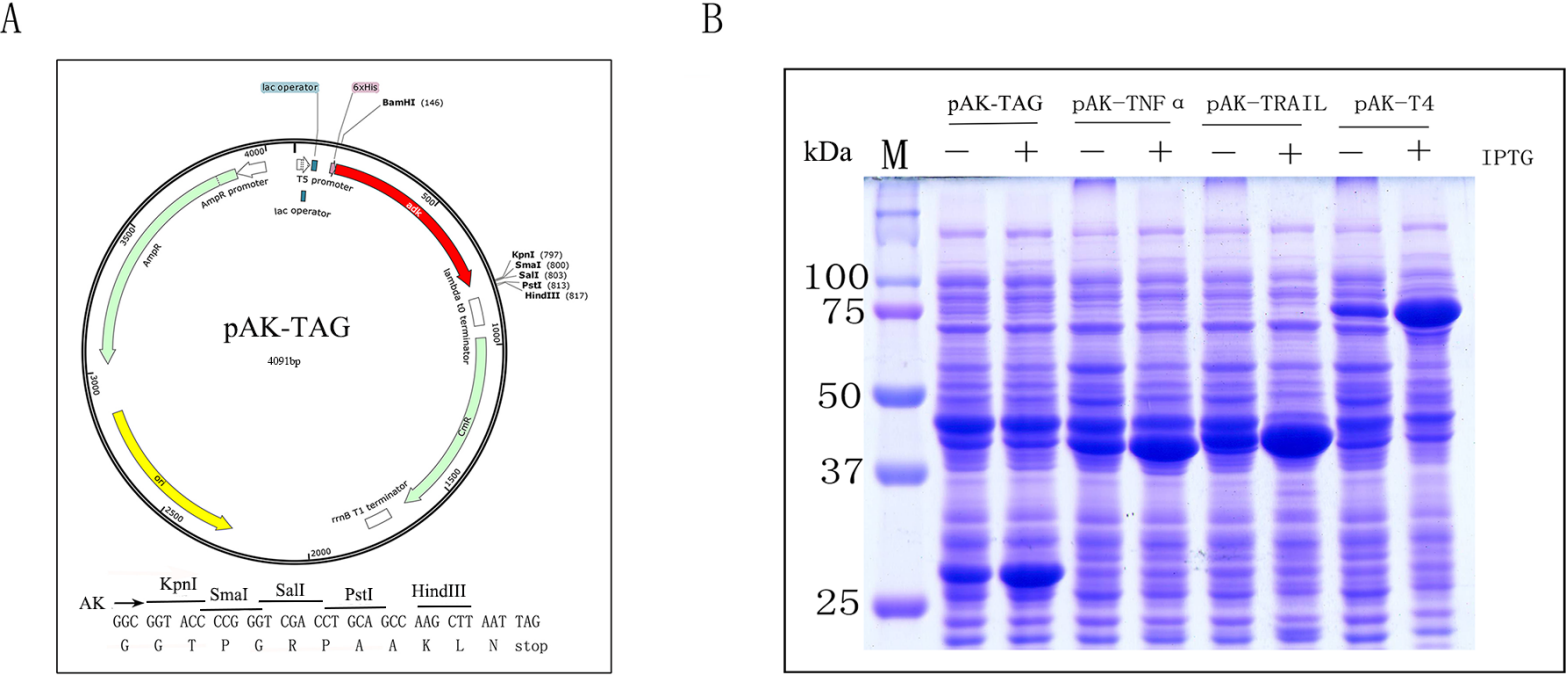
pAK-TAG expression vector and high level expression of recombinant AK fusion proteins in soluble form. (A) Schematic representation of the pAK-TAG vector. (B) SDS-PAGE analysis of the expression of AK-TNFα, AK-TRAIL, and AK-T4 DNA ligase.

We have previously described the expression of DNA ligase from bacteria phage T4 in soluble form as an AK fusion protein using pAK-TAG cloning vector we developed [30] (Fig 1A). pAK-TAG expression vector contains multiple cloning sites at the C-terminus of AK followed by a stop codon for convenient in-frame fusion of any recombinant protein. Under the control of a strong T5 promoter and the lac operator, the N-terminal His-tagged AK alone or any AK fusion proteins may be expressed at high level upon IPTG induction (Fig 1A) such as AK-T4 DNA ligase. To further demonstrate that the same goal can be attained for secreted proteins from mammalian cells, we have constructed several AK fusion proteins from human TNF family of cytokines. These fusion proteins, including AK-TNFα and AK-Trail as shown in Fig 1B, like T4 DNA ligase, were also expressed at high level in soluble forms upon IPTG induction. AK alone has a molecular weight at 27 kDa and both monomeric human TNFα and Trail have molecular weights around 16–17 kDa. The predicted molecular weights for AK-TNFα and AK-Trail thus would be around 43–44 kDa, which were consistent with our data presented (Fig 1B).

### One step purification of the AK-TNFα fusion protein by Blue-Sepharose with Ap5A affinity elution

**Fig 2:**
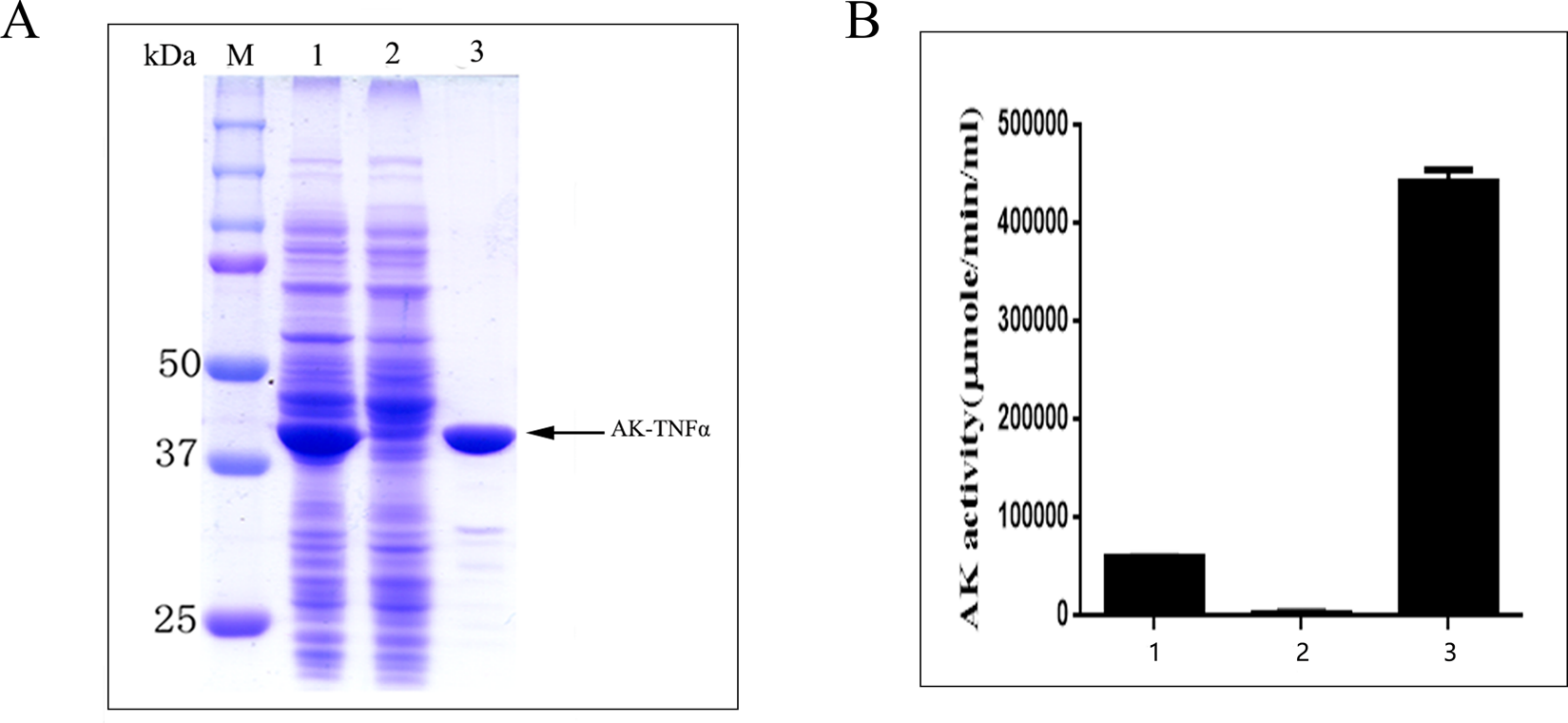
One step purification of AK-TNFα via Ap5A affinity elution off Blue-Sepharose chromatography. **(A)** SDS-PAGE analysis of AK-TNFα purification. M: Protein-molecular-weight size markers. Lane 1: Cleared soluble bacterial cell lysate. Lane 2: Flow-through fraction from Blue-Sepharose column. Lane 3: Ap5A elution of AK-TNFα from Blue-Sepharose. **(B)** AK enzyme activity during purification of AK-TNFα. 1: Cleared bacterial cell lysate. 2: Flow-through fraction from Blue-Sepharose column. 3: Ap5A elution of AK-TNFα from Blue-Sepharose. Data presented were representative of at least three independent experiments.

The original concept for using AK as an affinity tag was intended to take the advantage of its high-level bacterial expression, sensitive detection by enzyme assay and easy purification. However, the first AK fusion protein (AK-T4 DNA ligase) we expressed failed to come off the Blue-Sepharose column upon Ap5A elution due to the ability of T4 DNA ligase to bind to nucleotide-like dye moiety from the chromatographic matrix on its own [30]. We next chose proteins from mammalian cell origins that are known not to contain nucleotide or DNA binding properties, which are responsible for Blue-Sepharose binding. We showed that the AK-TNFα fusion protein behaved similarly to AK itself [26, 27] and was able to bind to Blue-Sepharose beads with little flow through. Upon complete washing off of any unbound proteins, AK-TNFα was efficiently and specifically eluted off the column by Ap5A with little contaminating proteins (Fig 2A). The specific binding of AK-TNFα to Blue-Sepharose and subsequent affinity elution with Ap5A was easily and quantitatively monitored in near real-time by AK enzyme activity during the purification (Fig 2B).

### Construction of pAK-TAG-T expression vector with thrombin cleavage site

**Fig 3:**
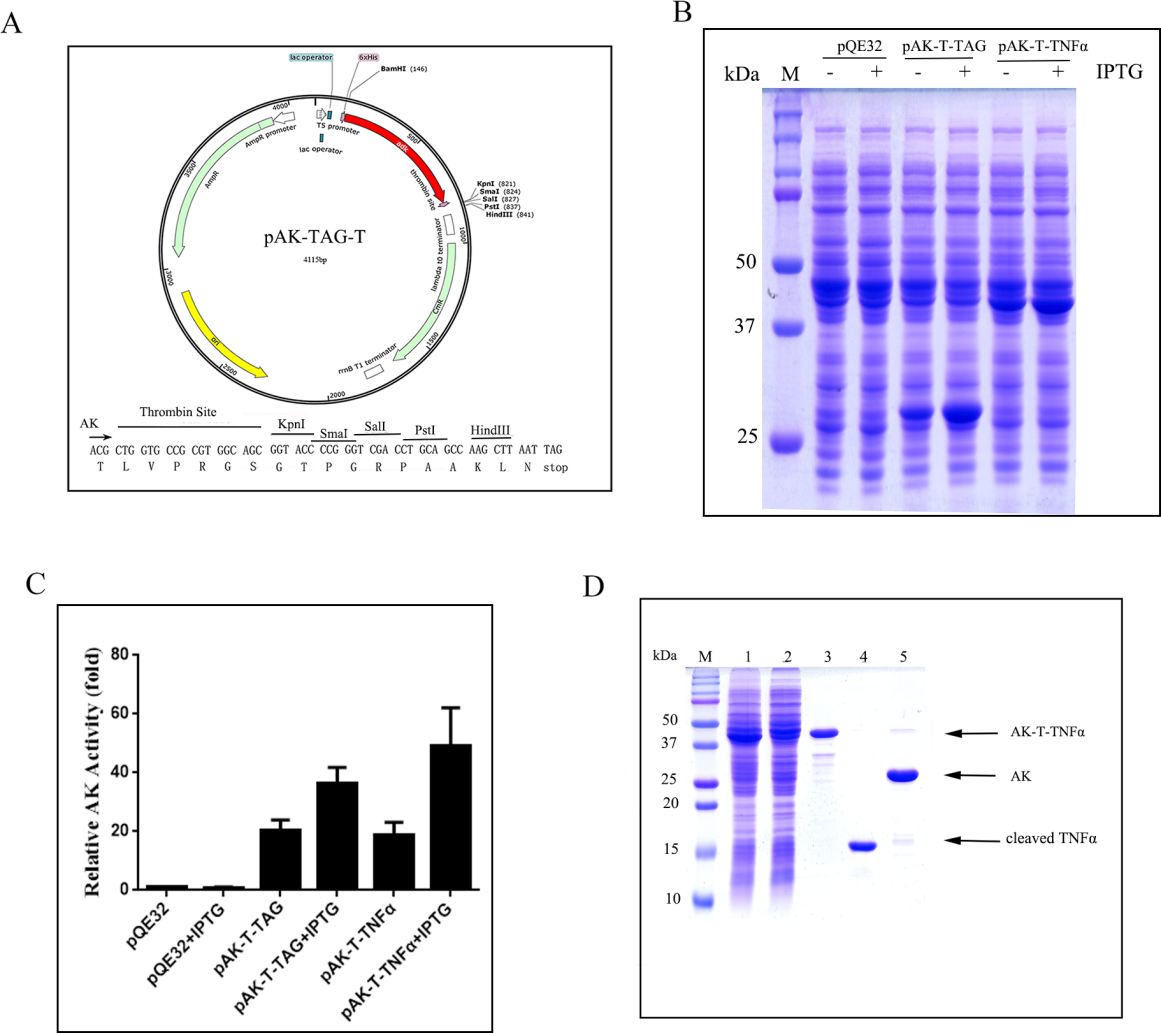
Construction of pAK-TAG-T expression vector with a thrombin cleavage site and the expression and purification of the cleaved TNFα. (A) Schematic representation of pAK-TAG-T expression vector. (B) SDS-PAGE analysis of the expression of AK-T-TNFα. (C) Estimate of the level of AK fusion protein expression by AK enzyme activity. (D) SDS-PAGE analysis the purification of the cleaved TNFα. AK-T-TNFα fusion protein was first purified via Ap5A affinity elution off a Blue-Sepharose column followed by purification of the cleaved TNFα off a Ni-NTA column upon thrombin digestion. M: Protein-molecular-weight size markers. Lane 1: Cleared soluble bacterial cell lysate. Lane 2: Flow-through fraction from Blue-Sepharose column. Lane 3: Ap5A Elution from Blue-Sepharose. Lane 4: Ni-NTA column and thrombin release of the cleaved TNFα. Lane 5: Imidazole elution of AK from the Ni-NTA column. Data presented were representative of at least three independent experiments.

To ensure that a cleaved recombinant protein could be recovered from its AK-fusion form, a thrombin cleavage site was introduced in-frame into the pAK-TAG expression vector before the multiple cloning sites. The resulting expression vector was designated as pAK-TAG-T (Fig 3A).

### High-level expression of AK-T-TNFα and estimation of its expression via AK activity

When human TNFα was cloned into pAK-TAG-T vector and transformed into XL1-blue, the resulting AK-T-TNFα fusion protein with a thrombin cleavage site in-between was expressed at high levels in soluble form upon IPTG induction, in comparison to AK expression from the pAK-TAG-T vector alone (Fig 3B). The level of AK-T-TNFα was easily measured via AK activity. Based on the increase in the specific activity of AK, AK-T-TNFα expression level was estimated to be over 50 times of endogenous AK from the host cells (Fig 3C). Due to the high copy number of the pAK-TAG-T vector and strong T5 promoter, there were leaky expression of AK-T and AK-T fusion proteins without the inducer.

The one-step purifications of AK-TNFα and AK-T-TNFα via Ap5A affinity elution were easily monitored by the AK activity of the fusion proteins. The yield of recovery was estimated to be around 50% with over 7-fold purification for both AK-TNFα **(**Fig 2) and AK-T-TNFα **(**Fig 3) fusion proteins based on the increase in specific activity of AK (Table 1). SDS-PAGE analysis followed by densitometry quantification indicated that about 95% purity was achieved after Ap5A affinity elution. In terms of the yield of purified AK fusion proteins based on bacterial culture, it translated to about 18 and 17 mg/L for AK-TNFα and AK-T-TNFα, respectively.

**Table 1:** Purification Chart of AK-TNFα and AK-T-TNFα from 300 ml of Bacterial Culture*.

**AK-TNFα**					
Purification Step	Total Protein (mg)	Total AK Activity (U)	Specific Activity (U/mg)	Fold of Purification	Yield (%)
Cell Lysate	77.9	4.6x10^6^	5.9x10^4^	1.0	100
Blue-Sepharose	5.4	2.3x10^6^	4.4x10^5^	7.5	50.5
**AK-T-TNFα and Cleaved TNFα**						
Purification Step	Total Protein (mg)	Total AK Activity (U)	Specific Activity (U/mg)	Fold of Purification	Yield (%)
Cell Lysate	74.7	5.8x10^6^	7.8x10^4^	1.0	100
Blue-Sepharose	5.1	2.8x10^6^	5.4x10^5^	7.0	47.3
Thrombin Cleaved TNFα	1.9	–	–	–	44.0

* Results were representative of at least 2 independent experiments.

### Two step purification of cleaved human TNFα

Like described above, the AK-T-TNFα fusion protein with a thrombin cleavage site was first purified to near homogeneity via Blue-Sepharose with Ap5A elution (Fig 3D). To release the cleaved TNFα from AK, the purified AK-T-TNFα fusion protein was then loaded onto a Ni-NTA column. Because the AK was N-terminal His-tagged, the AK-T-TNFα was captured by the Ni-NTA. Then thrombin was added to allow in-column cleavage of the fusion protein overnight. The cleaved human TNFα was then released from the Ni-NTA column with the elution buffer with low concentration of imidazole, while the bound AK moiety was later eluted with a linear-gradient of 5–500 mM imidazole (Fig 3D). The in-column thrombin release of TNFα was estimated to be 93% in step-yield based on the protein concentration and molecular mass ratio of TNFα in AK-T-TNFα, which gave rise to an overall yield of recovery over 44% (Table 1) with about 6.3 mg/L of TNFα from bacteria cell culture.

### Characterization of purified human TNFα and AK-TNFα

**Fig 4:**
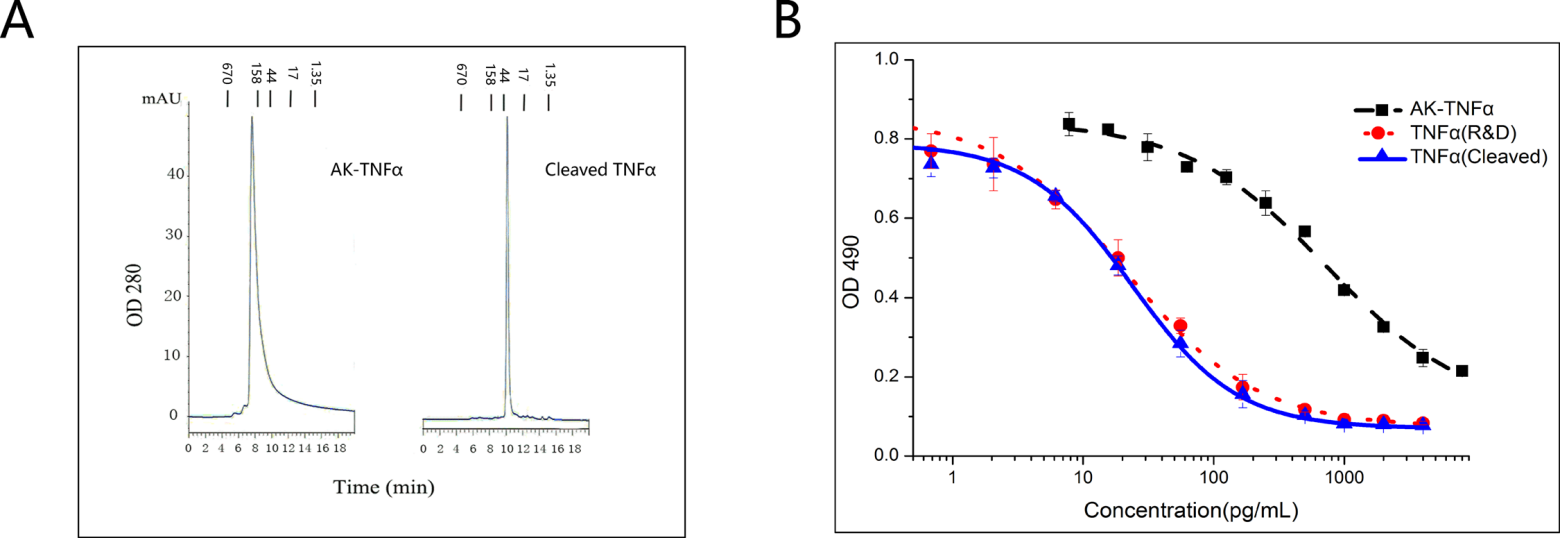
Characterization of the purified AK-TNFα and cleaved TNFα. (A) SEC-HPLC analysis of the purity and estimation of the molecular-weight of AK-TNFα and TNFα. (B) Analysis of the biological function (L929 assay) and determination of the IC50 of AK-TNFα and cleaved TNFα in comparison to a commercial source of TNFα (R&D Systems). Data presented were representative of at least three independent experiments.

TNFα family of cytokines are all trimeric in structure which is required for their full biological functions. To determine the conformation of AK-TNFα fusion protein and the cleaved TNFα purified with our AK-TAG method, we analyzed them on a size exclusion HPLC (SEC-HPLC). Both purified AK-TNFα and the cleaved TNFα showed as single peaks with estimated molecular weight consistent with being trimers (Fig 4A). Functionally, the AK-TNFα fusion protein had not only the AK activity but also retained TNFα biological function as determined by its cytotoxicity to L929 cells [32](Fig 4B). However, its potency was over 13 times less than that of the cleaved TNFα upon being released from thrombin cleavage (Fig 4B). The cleaved TNFα generated with our AK-TAG method exhibited the comparable potency in IC50 in L929 bioassay to TNFα from R&D systems (Table 2).

**Table 2:** Bioactivity of AK-TNFα and TNFα.*

	TNFα (R&D)	TNFα (cleaved)	AK-TNFα
IC50 (pg/mL)	22.6±3.0	22.8±3.4	737.8±79.8
IC50 (pM)	1.4±0.2	1.4±0.2	17.4±1.9

^*^ Results were representative of at least 3 independent experiments.

## Discussion

In this study we demonstrated that multiple mammalian proteins could be expressed at high level in soluble forms with AK-TAG. The functional dye ligand from Blue-Sepharose is Cibacron Blue F3G-A. Some proteins and enzymes such as AK interact biospecifically with the dye due to its structural similarity with nucleotide cofactors or substrates, while others, such as albumin and interferon, bind in a less specific manner by electrostatic and/or hydrophobic interactions with the aromatic anionic ligand [33]. We showed that the AK-TNFα fusion protein behaved similarly to AK alone and could be efficiently purified to near homogeneity in a single chromatographic step using Blue-Sepharose via Ap5A affinity elution. Ap5A, which is a transition state substrate analog of AK [27], binds to *E coli* AK with high affinity and enabled the complete elution of AK-TNFα at μM range. This is in contrast to eluents used for other affinity tags, which often require eluents several orders of magnitude higher in concentration such as imidazole (for His-tag). Such a high specificity of Ap5A to AK ensures an unparalleled purity achievable with AK-TAG. We routinely achieved the final yield of highly purified AK-TNF-α in the range of 17–18 mg/L within 2–3 days starting from the seed culture. Although higher yield in commercial production for TNFα had been reported previously, the process employed two weeks of high-density bacteria fermentation [34] instead of shake-flask process that we used.

As noted above, some target proteins, unlike TNFα, may bind to Blue-Sepharose on their own in a less specific manner via electrostatic and/or hydrophobic interactions with the aromatic anionic ligand. In these cases, such AK fusion proteins may not be eluted by Ap5A alone and require less specific elution with a proper salt concentration such as in the case of AK-T4 DNA ligase fusion protein [30]. Although salt elution is less specific, remaining impurity may be easily and completely removed via a subsequent Ni-NTA column with imidazole elution, by taking the advantage of a 6xHis-tag engineered at the N-terminus of AK [30].

Another advantage of using AK as both a solubility and an affinity tag for recombinant protein expression and purification is its highly active enzyme activity and a relative easy assay. The extent of soluble expression of a recombinant AK fusion protein can be quickly and quantitatively assessed by AK enzyme activity assay in comparison with that of the host cells, which can be achieved within minutes, in contrast to the lengthy SDS-PAGE analysis. Also, an AK fusion protein may be monitored in near real time by the AK activity during the purification, which can allow yield of recovery to be easily for each purification step [30, 31].

For any affinity tag used for a recombinant protein production, it is often desirable to have the option of having the tag being removed in order to obtain a cleaved target protein with a full biological function[14, 16]. To this end, we introduced a thrombin cleavage site at the C-terminus of AK to obtain the pAK-TAG-T expression vector. This allows us to purify the cleaved TNFα protein to homogeneity with ease by a Blue-Sepharose column via Ap5A elution followed by in-gel thrombin release on a Ni-NTA column, while AK-TAG remains bound to the matrix though a 6xHis-tag. Thus, AK-TAG is a highly streamlined method for high-level soluble expression and fine purification of recombinant proteins. It complements other expression vectors with different affinity tags and is particularly useful when high yield and purity of a cleaved recombinant protein is desired.

## References

[1] Nallamsetty S, Waugh DS. A generic protocol for the expression and purification of recombinant proteins in Escherichia coli using a combinatorial His6-maltose binding protein fusion tag. Nature protocols. 2007;2(2):383–91. 10.1038/nprot.2007.50 . 17406599

[2] Graslund S, Nordlund P, Weigelt J, Bray J, Hallberg BM, Gileadi O, et al Protein production and purification. Nature methods. 2008;5(2):135–46. 10.1038/nmeth.f.202 18235434 PMC3178102

[3] McCluskey AJ, Poon GM, Gariepy J. A rapid and universal tandem-purification strategy for recombinant proteins. Protein science: a publication of the Protein Society. 2007;16(12):2726–32. 10.1110/ps.072894407 17965191 PMC2222826

[4] Young CL, Britton ZT, Robinson AS. Recombinant protein expression and purification: a comprehensive review of affinity tags and microbial applications. Biotechnology journal. 2012;7(5):620–34. 10.1002/biot.201100155 . 22442034

[5] Lebendiker M, Danieli T. Production of prone-to-aggregate proteins. FEBS letters. 2014;588(2):236–46. 10.1016/j.febslet.2013.10.044 . 24211444

[6] Esposito D, Chatterjee DK. Enhancement of soluble protein expression through the use of fusion tags. Current opinion in biotechnology. 2006;17(4):353–8. 10.1016/j.copbio.2006.06.003 . 16781139

[7] Goulas T, Cuppari A, Garcia-Castellanos R, Snipas S, Glockshuber R, Arolas JL, et al The pCri System: a vector collection for recombinant protein expression and purification. PloS one. 2014;9(11):e112643 10.1371/journal.pone.0112643 25386923 PMC4227841

[8] Nallamsetty S, Austin BP, Penrose KJ, Waugh DS. Gateway vectors for the production of combinatorially-tagged His6-MBP fusion proteins in the cytoplasm and periplasm of Escherichia coli. Protein science: a publication of the Protein Society. 2005;14(12):2964–71. 10.1110/ps.051718605 16322578 PMC2253240

[9] Waugh DS. Making the most of affinity tags. Trends in biotechnology. 2005;23(6):316–20. 10.1016/j.tibtech.2005.03.012 . 15922084

[10] Abdelhamid MA, Motomura K, Ikeda T, Ishida T, Hirota R, Kuroda A. Affinity purification of recombinant proteins using a novel silica-binding peptide as a fusion tag. Applied microbiology and biotechnology. 2014;98(12):5677–84. 10.1007/s00253-014-5754-z . 24756322

[11] Smith DB, Johnson KS. Single-step purification of polypeptides expressed in Escherichia coli as fusions with glutathione S-transferase. Gene. 1988;67(1):31–40. Epub 1988/07/15. . 3047011 10.1016/0378-1119(88)90005-4

[12] Maina CV, Riggs PD, Iii AGG, Slatko BE, Moran LS, Tagliamonte JA, et al An Escherichia coli vector to express and purify foreign proteins by fusion to and separation from maltose-binding protein. Gene. 1988;74(2):365–73. 3073105 10.1016/0378-1119(88)90170-9

[13] Hochuli E, Bannwarth W, Döbeli H, Gentz R, Stüber D. Genetic approach to facilitate purification of recombinant proteins with a novel metal chelate adsorbent. Nature biotechnology. 1988;6(11):1321–5.

[14] Fong BA, Wu WY, Wood DW. The potential role of self-cleaving purification tags in commercial-scale processes. Trends in biotechnology. 2010;28(5):272–9. 10.1016/j.tibtech.2010.02.003 . 20359761

[15] Lawrence D, Shahrokh Z, Marsters S, Achilles K, Shih D, Mounho B, et al Differential hepatocyte toxicity of recombinant APO2L/TRAIL versions. Nature medicine. 2001;7(4):383–5. 11283636 10.1038/86397

[16] Eschenfeldt WH, Maltseva N, Stols L, Donnelly MI, Gu M, Nocek B, et al Cleavable C-terminal His-tag vectors for structure determination. Journal of structural and functional genomics. 2010;11(1):31–9. 10.1007/s10969-010-9082-y 20213425 PMC2885959

[17] Frey S, Gorlich D. A new set of highly efficient, tag-cleaving proteases for purifying recombinant proteins. Journal of chromatography A. 2014;1337:95–105. 10.1016/j.chroma.2014.02.029 . 24636565

[18] Chang JY, Alkan SS, Hilschmann N, Braun DG. Thrombin specificity. Selective cleavage of antibody light chains at the joints of variable with joining regions and joining with constant regions. European journal of biochemistry / FEBS. 1985;151(2):225–30. Epub 1985/09/02. . 3928376 10.1111/j.1432-1033.1985.tb09092.x

[19] Di Cera E, Dang QD, Ayala YM. Molecular mechanisms of thrombin function. Cellular and molecular life sciences: CMLS. 1997;53(9):701–30. Epub 1997/11/22. . 9368668 10.1007/s000180050091 PMC11147250

[20] Hefti MH, Van Vugt-Van der Toorn CJ, Dixon R, Vervoort J. A novel purification method for histidine-tagged proteins containing a thrombin cleavage site. Analytical biochemistry. 2001;295(2):180–5. 10.1006/abio.2001.5214 . 11488620

[21] Terpe K. Overview of tag protein fusions: from molecular and biochemical fundamentals to commercial systems. Applied microbiology and biotechnology. 2003;60(5):523–33. 10.1007/s00253-002-1158-6 . 12536251

[22] Hwang PM, Pan JS, Sykes BD. Targeted expression, purification, and cleavage of fusion proteins from inclusion bodies in Escherichia coli. FEBS letters. 2014;588(2):247–52. 10.1016/j.febslet.2013.09.028 . 24076468

[23] Bornhorst JA, Falke JJ. Purification of proteins using polyhistidine affinity tags. Methods in enzymology. 2000;326:245–54. Epub 2000/10/19. ; PubMed Central PMCID: PMCPmc2909483. 11036646 10.1016/s0076-6879(00)26058-8 PMC2909483

[24] Kupriyanov VV, Ferretti JA, Balaban RS. Muscle adenylate kinase catalyzes adenosine 5'-tetraphosphate synthesis from ATP and ADP. Biochimica et biophysica acta. 1986;869(1):107–11. Epub 1986/01/17. . 3002476 10.1016/0167-4838(86)90316-x

[25] Liang P, Glaser M. Efficient cloning of a mutant adenylate-kinase-encoding gene from Escherichia coli. Gene. 1989;80(1):21–8. Epub 1989/08/01. . 2551784 10.1016/0378-1119(89)90246-1

[26] Barzu O, Michelson S. Simple and fast purification of Escherichia coli adenylate kinase. FEBS letters. 1983;153(2):280–4. Epub 1983/03/21. . 6311616 10.1016/0014-5793(83)80624-3

[27] Liang P, Phillips GN Jr., Glaser M. Assignment of the nucleotide binding sites and the mechanism of substrate inhibition of Escherichia coli adenylate kinase. Proteins. 1991;9(1):28–36. Epub 1991/01/01. 10.1002/prot.340090105 . 2017434

[28] Lienhard GE, Secemski II. P 1, P 5 -Di(adenosine-5')pentaphosphate, a potent multisubstrate inhibitor of adenylate kinase. The Journal of biological chemistry. 1973;248(3):1121–3. Epub 1973/02/10. . 4734335

[29] Huss RJ, Glaser M. Identification and purification of an adenylate kinase-associated protein that influences the thermolability of adenylate kinase from a temperature-sensitive adk mutant of Escherichia coli. The Journal of biological chemistry. 1983;258(21):13370–6. Epub 1983/11/10. . 6313692

[30] Liu X, Huang A, Luo D, Liu H, Han H, Xu Y, et al Use of adenylate kinase as a solubility tag for high level expression of T4 DNA ligase in Escherichia coli. Protein Expr Purif. 2015;109:79–84. 10.1016/j.pep.2015.02.010 . 25700573

[31] Ma Z, Luo D, Huang A, Xu Y, Wang Y, Wei Y, et al pKILLIN: a versatile positive-selection cloning vector based on the toxicity of Killin in Escherichia coli. Gene. 2014;544(2):228–35. 10.1016/j.gene.2014.04.037 . 24768186

[32] Shiau MY, Chiou HL, Lee YL, Kuo TM, Chang YH. Establishment of a consistent L929 bioassay system for TNF-alpha quantitation to evaluate the effect of lipopolysaccharide, phytomitogens and cytodifferentiation agents on cytotoxicity of TNF-alpha secreted by adherent human mononuclear cells. Mediators Inflamm. 2001;10(4):199–208. Epub 2001/10/02. 10.1080/09629350123139 ; PubMed Central PMCID: PMCPmc1781708. 11577996 PMC1781708

[33] Thompson ST, Cass KH, Stellwagen E,. Blue dextran-sepharose: an affinity column for the dinucleotide fold in proteins. Proceedings of the National Academy of Sciences of the United States of America. 1975;72(2):669–72. 164664 10.1073/pnas.72.2.669 PMC432376

[34] Corti A. Tumor necrosis factor: methods and protocols: Springer Science & Business Media; 2004.

